# A quantitative approach to the spread of variance in translational research using Monte Carlo simulation

**DOI:** 10.1038/s41598-022-09921-3

**Published:** 2022-04-15

**Authors:** Feyza Cukurova, Britta P. Gustavson, Andres G. Griborio-Guzman, Leonard A. Levin

**Affiliations:** 1 Department of Ophthalmology, McGill University, Montreal, Canada; 2 Department of Neurology & Neurosurgery, McGill University, Montreal, Canada; 3 Montreal Neurological Institute, 3801 University Ave, Montreal, QC H3A 2B4 Canada

**Keywords:** Drug development, Translational research

## Abstract

The translation of promising preclinical research into successful trials often fails. One contributing factor is the “Princess and the Pea” problem, which refers to how an initially significant effect size dissipates as research transitions to more complex systems. This work aimed to quantify the effects of spreading variability on sample size requirements. Sample size estimates were performed by Monte Carlo simulation. To simulate the process of progressing from preclinical to clinical studies, nested sigmoidal dose–response transformations with modifiable input parameter variability were used. The results demonstrated that adding variabilty to the dose–response parameters substantially increases sample size requirements compared to standared calculations. Increasing the number of consecutive studies further increases the sample size. These results quantitatively demonstrate how the spread of variability in translational research, which is not typically accounted for, can result in drastic increases in the sample size required to maintain a desired study power.

## Introduction

The translation of preclinical studies of a novel therapeutic into successful phase 2 and 3 trials is a process that frequently fails, despite high-quality basic research and well-conducted clinical studies. The literature is replete with examples of such translational failures, including attempts to bring drugs for septic shock to the market, the development of neuroprotective therapies for stroke and glaucoma, and vaccines for human immunodeficiency virus (HIV).

We previously outlined three structural reasons to explain this *Lost in Translation* phenomenon^1^. The first, the *Butterfly Effect*, refers to how minute differences between preclinical models can result in significantly different results. For instance, two different methods of increasing intraocular pressure in rat models of glaucoma can result in significant differences in the ability of the same neuroprotective agent to protect the optic nerve^2,3^. If minor “input” differences between relatively homogenous animal models produce such vast “output” effects, it is unsurprising that translating preclinical animal work into human studies frequently fails. Using ensembles of animal models was proposed as a strategy to combat the chaos inherent in biological systems and thereby increase the likelihood of a successful bridge between preclinical and clinical studies^4^.

The second factor contributing to failed translation is the concept of the *Two Cultures*. This concept highlights the differences in how experiments are designed, analyzed, and executed in the setting of preclinical research, compared to that of clinical trials. One solution for the Two Cultures problem is to establish harmonization and communication of the experimental process among preclinical and clinical researchers^4^.

The third factor, and the central focus of the current study, is the *Princess and the Pea* problem^1^. In the eponymous Hans Christian Andersen tale, a weatherworn princess proves her nobility by having trouble sleeping due to of a pea at the bottom of a stack of mattresses. In the real world of scientific research, a pea-sized biological effect is effectively null after a series of sequential experiments; this is in large part due to variability. The Princess and the Pea problem specifically refers to the accumulation of variability as research is carried out along the developmental pathway comprising the molecular, receptor, intracellular messaging, tissue, animal, and eventually clinical trial levels. As variability accumulates, the effect size of the intervention gradually becomes lost in the noise, akin to how the bump from a pea (or even a rock) becomes lost in the padding of a pile of mattresses. The critical issue underlying the Princess and the Pea problem is that it is so intrinsic to the translational research process, particularly that of drug discovery, that quantifying its effect and proposing a solution has been elusive.

There are several reasons why variability increases as research transitions from molecular to animal to human studies. For example, a novel therapeutic agent can be first shown to bind its receptor under highly controlled chemical reaction conditions. Variability is here at its lowest. The move to cell culture, where conditions remain tightly controlled, introduces more variability as a reflection of the many ongoing metabolic reactions occurring within a living cell. Animal studies carry even higher levels of variability. Despite the standard of comparing inbred animals of the same sex and age, there are many factors that add variability to animal studies. Genetic variability has been shown to persist even in inbred animals^5^, experimental animals demonstrate epigenetic differences^6,7^, and the impact of husbandry, housing, interactions with experimenters, pheromones, and the animal’s microbiome all contribute to the variability of animal models^8,9^.

As the drug development process moves to humans, researchers encounter even more variability. Human subjects are rarely genetically identical, and epigenetic differences between humans are effectively impossible to avoid. Moreover, clinical trial participants vary in the time they take to become symptomatic for a given condition, when they seek treatment, their compliance with the treatment, the degree of placebo effect, and their previous medical history^1^.

The vast majority of translational studies rely on determining if the mean difference between groups, divided by some measure of variability, produces a value that is statistically significant. The observed spread of variance as more complex chemical and biological systems are studied causes this ratio to decrease, essentially reducing the effect relative to the variability. Even if the effect size stays the same, an increased variance makes it increasingly challenging to detect differences between experimental groups. If one adds distributions that have specific variances, the variance of the sum will increase. The variance of the sum or difference of independent random variables is equal to the sum of its variances, i.e., variation will increase with addition or subtraction of these distributions. In other words, the effects of progressive experiments, from simple reactions to more complex biological systems, each with its own variability, will increase the overall variability of a study. This is at the heart of the Princess and the Pea problem.

To quantify the effects of the Princess and the Pea problem in the biomedical arena we performed a series of Monte Carlo simulations. Sigmoidal dose–response curves were selected as example transfer functions, each adding variability based on their parameters, and used to calculate study sample size requirements. This approach was used to quantify the impact of introducing different amounts of variability on a single experiment and series of experiments, making possible the ability to use the effect size in an animal model to an estimated size for a clinical trial. In some cases, a realistic degree of variability in a series of simulated experiments could result in a situation where a clinical trial is impossible because of an impractically large study size needed to detect a significant difference between groups.

## Results

**Figure 1:**
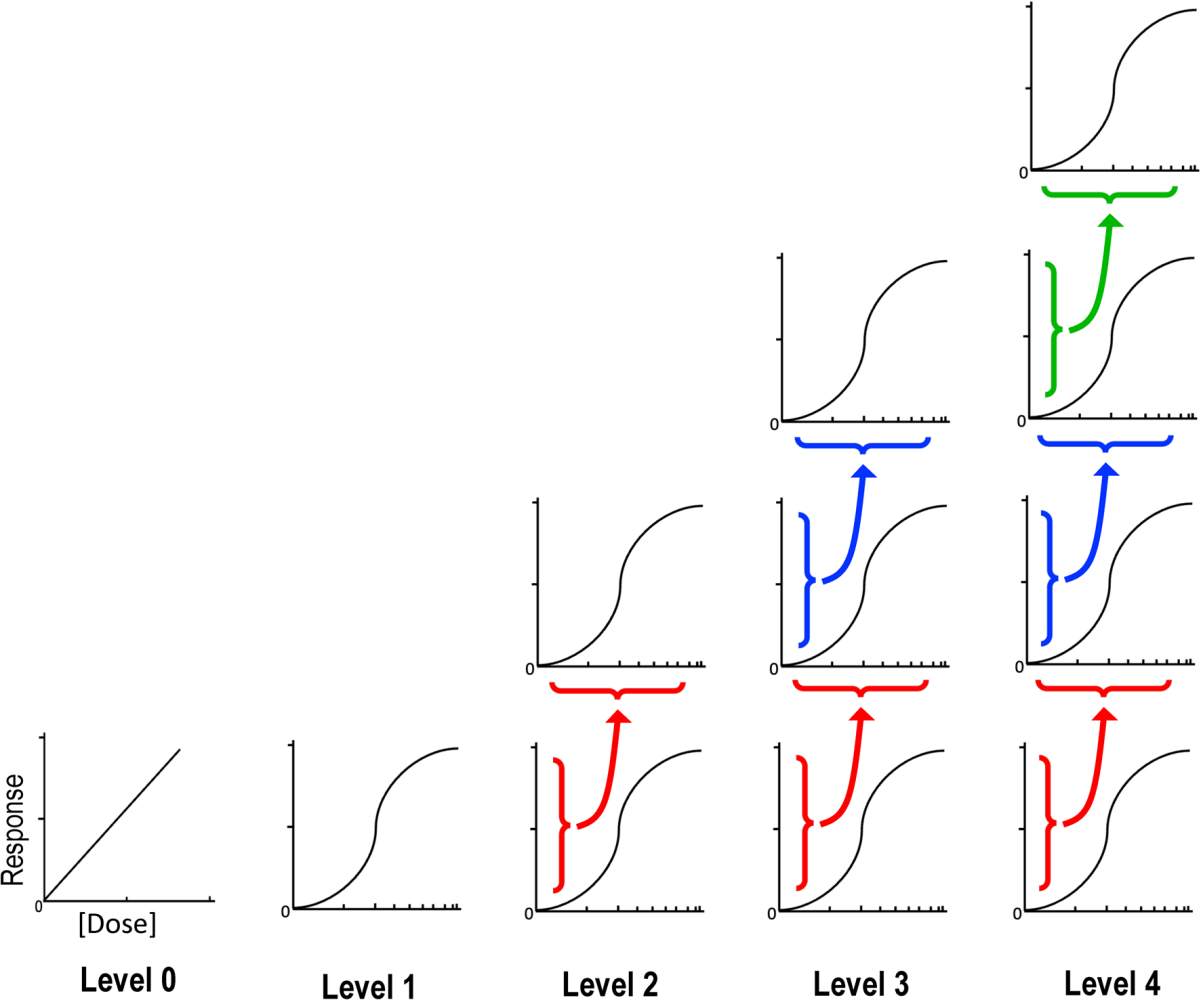
Modeling the sequence of drug effect with series of dose–response transformations. Level 0 models a linear relationship between drug dose and response. Level 1 transforms dose parameter vectors by the sigmoidal Hill dose–response function. Level 2 transforms Level 1 response outcomes by a second sigmoidal dose–response function. Level 3 uses three consecutive dose–response functions, using the response output from one level as the dose input for the next. Level 4 uses four consecutive dose–response functions.

The following results quantify the impact of simulating consecutive experiments (Fig. 1) and/or adding variability to the parameters of each experiment on sample size requirements and, correspondingly, study feasibility. Each Level, described below, represents a study along the pathway from pre-clinical work to late-phase clinical trials.

The simulated experiments are based on nested dose–response transformations, where each level represents an additional dose response transformation. The following terminology has been used: Level 0: no dose response transformation; Level 1: one dose–response transformation (i.e. one study); Level 2: two consecutive dose–response transformations (i.e. one study building on the work of a previous study); Level 3: three consecutive dose–response transformations; Level 4: four consecutive dose–response transformations. For the consecutive dose–response transformations, the response output from one transformation was used as the dose input data for the subsequent transformation, as outlined in Fig. 1.

### Power to detect differences in two normal distributions transformed with a dose–response curve

We first established the validity of the Monte Carlo simulation for comparison of two samples by assessing the power calculations from the simulation versus those calculated by standard methods based on the non-central *t* distribution. To do this, two vectors of n normally distributed values differing in their means were generated, simulating a single experiment. An unpaired equal-variance *t*-test was performed between the two vectors, and the trial was recorded as significant if the p value was less than the predetermined alpha (0.05). This process was repeated 10,000 times, based on initial simulations demonstrating significantly more variable responses with 1000 runs but low variability at 10,000 or 100,000 runs. The n required to obtain a specific power (i.e. proportion of trials that showed a significant difference) for the predetermined difference of means was determined by adjusting n and repeating the simulation. Finally, the simulated power was compared to the power calculated using the MATLAB *sampsizepwr* function for a *t*-test. The results of these comparisons demonstrated that the simulation replicated the same relationship among study power, sample size, and group mean difference as with MATLAB functions.

**Figure 2:**
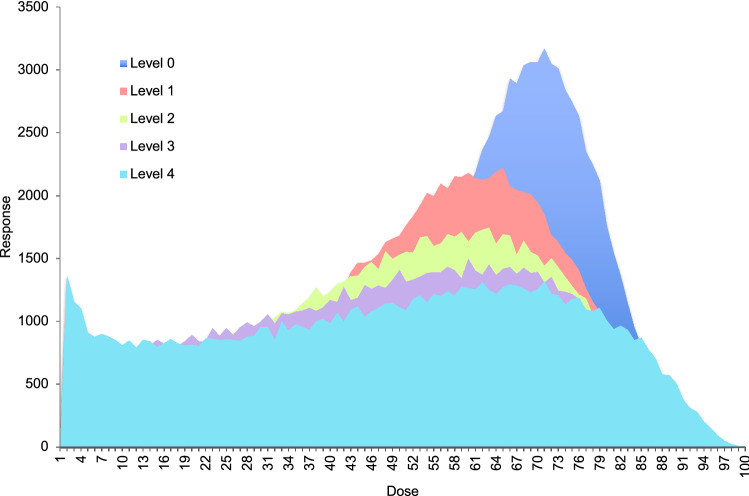
Multiple spreads of variance. Sequential sigmoidal dose–response transformations of a normally distributed dataset (blue) increase the spread of variance among experimental data for an arbitrary dose–response simulation of consecutive sigmoidal dose–response functions, with greater spread with each number of transformation (dark blue = no dose–response transformation/Level 0, red = level 1, green = level 2, purple = level 3, turquoise = level 4).

The process of transforming data through dose–response functions, even without additional variability in the parameters, can be shown to increase the noisiness or spread of the data (Fig. 2). When an arbitrary, normally-distributed drug dose concentration is run through 1, 2, 3, or 4 dose–response transformations, there is a gradual widening of the initially normal distribution.

**Figure 3:**
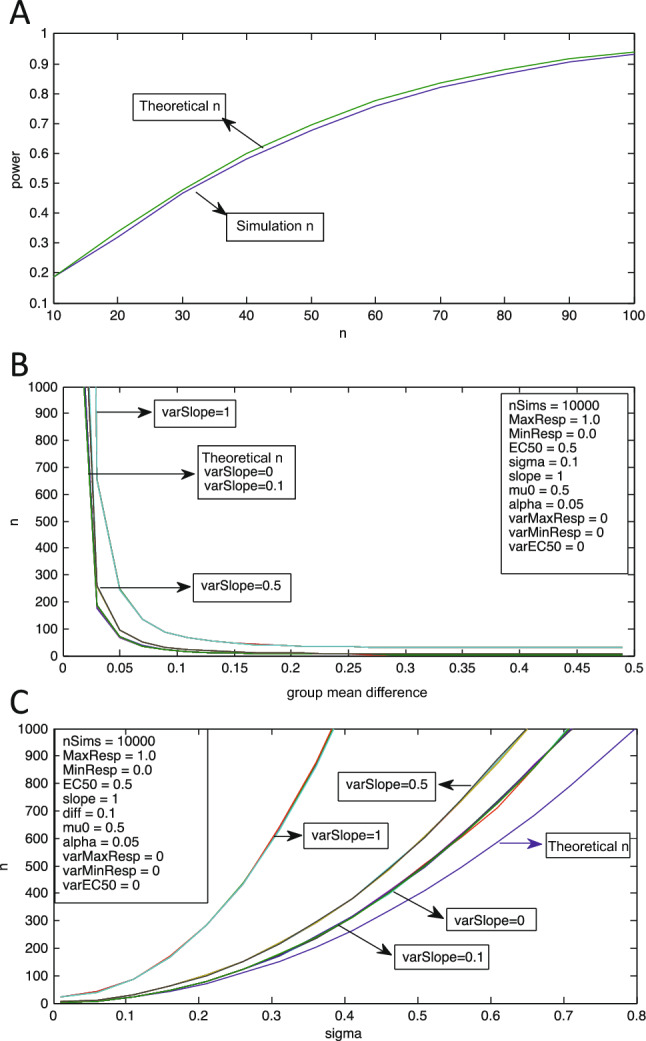
(**A**) Effect of sigmoidal dose–response transformation on power for various sample sizes. The simulation power analysis based on transformed dose–response data (simulation power) is slightly less than the power analysis based on MATLAB calculations using the *sampsizepwr* function (theoretical power). The theoretical power analysis does not take into account a dose–response transformation. Group mean difference = 0.1, sigma = 0.2, EC_50_ = 0.5, slope = 1, MaxResp = 1, MinResp = 0, alpha = 0.05, nSimulations = 10,000. (**B**) Effect of slope variability [varSlope; Formula (2)] on sample size for Level 2 simulations at various group mean differences. The difference between the simulation sample size estimates and the theoretical sample size increases at a given group mean difference when greater variability is added to the slope parameter by increasing the varSlope value, with alpha and power held constant. Note that the curves for “Theoretical n,” “varSlope = 0,” and “varSlope = 0.1” are virtually identical and are indicated together. (**C**) Similar to (**B**) but for various sigma values.

On the basis of this observation, a dose–response curve transformation was added to the simulation and the effect on power and sample size assessed. This constituted Level 1 (equivalent to the blue distribution in Fig. 2). To do this, two new vectors for comparison of groups were produced by transforming the dose parameter input vectors (equivalent to “doses”), each drawn from normal distributions differing in means, and applying a Hill dose–response function. The simulations were run as above, again using various parameters to assess for their effect on power. For example, in Fig. 3A the difference of means was set to 0.1, the SD of the input distribution (sigma) to 0.2, EC_50_ to 0.5, slope to 1, maximal response to 1, and minimal response to 0. Using these parameters, there was minimal difference between the power analysis based on the transformed (dose response) data and that based on the untransformed data. For all data, theoretical power and theoretical sample size refer to values calculated using standard MATLAB methods, as opposed to using the Monte Carlo simulation. The largest discrepancy for those simulations was where *n* ranged from 38 to 67. At an *n* of 100 the simulation power closely approximated the theoretical power, with both power estimates keeping with a desired study power (power of 0.933 vs. 0.941, respectively). These data demonstrate that the simulation replicates MATLAB t-test power calculations when the input data are transformed by the dose–response relationship (Level 1). Above an *n* of 67, the greater the sample size, the closer the simulation is to providing the same power estimates as a standard MATLAB power function.

Third, the effect of a second level of dose–response transformation was simulated, i.e., the output of one dose–response transformation was used as input for a second transformation (Fig. 1, Level 2). As with Level 1, there was no substantial change in power and sample size calculations for Level 2 when compared to power and sample size calculated by *sampsizepwr* in MATLAB when no variability was added to the dose–response parameters. For example, at a group mean difference of 0.11, a SD of 0.1 and a slope variance of 0, the simulated sample size and the theoretical sample size are both 15 (Table 1; Fig. 3B, varSlope = 0 curve). Only when the group mean difference is very small (e.g., 0.01) does stepping up a Level (adding an additional dose–response transformation) without adding variability result in an appreciable difference between the two estimates, with a simulated sample size of 1643 and a theoretical sample size of 1571 (Table 1).

**Table 1:** Sample size requirements at Levels 2 and 3, based on adding variability to each individual dose–response curve parameter.

	Sample size (n)				
	Theoretical	Slope	EC50	MaxResp	MinResp
**Level 2**					
Group mean difference = 0.01					
Variability					
0.0	1571	1643 (1.05x)	1636 (1.04x)	1657 (1.05x)	1658 (1.06x)
0.1	1571	1666 (1.06x)	3467 (2.21x)	2016 (1.28x)	2103 (1.28x)
0.5	1571	2298 (1.46x)	> 10,000 (6.37x)	> 10,000 (6.37x)	> 10,000 (6.37x)
1.0	1571	5743 (3.66x)	> 10,000 (6.37x)	> 10,000 (6.37x)	> 10,000 (6.37x)
Group mean difference = 0.11					
Variability					
0.0	15	15 (1.00x)	15 (1.00x)	15 (1.00x)	15 (1.00x)
0.1	15	16 (1.07x)	35 (2.33x)	20 (1.33x)	19 (1.27x)
0.5	15	22 (1.47x)	679 (45x)	163 (11x)	118 (7.87x)
1.0	15	66 (4.40x)	3313 (221x)	885 (59x)	692 (46x)
**Level 3**					
Group mean difference = 0.01					
Variability					
0.0	1571	1708 (1.09x)	1708 (1.09x)	1714 (1.09x)	1714 (1.09x)
0.1	1571	1741 (1.11x)	4117 (2.62x)	2181 (1.39x)	2331 (1.48x)
0.5	1571	2652 (1.69x)	> 10,000 (6.37x)	> 10,000 (6.37x)	> 10,000 (6.37x)
1.0	1571	> 10,000 (6.37x)	> 10,000 (6.37x)	> 10,000 (6.367x)	> 10,000 (6.37x)
Group mean difference = 0.11					
Variability					
0.0	15	16 (1.07x)	16 (1.07x)	16 (1.07x)	16 (1.07x)
0.1	15	17 (1.13x)	43 (2.87x)	23 (1.53x)	22 (1.47x)
0.5	15	26 (1.73x)	1108 (74x)	267 (18x)	233 (16x)
1.0	15	135 (9.00x)	3456 (230x)	2159 (144x)	2916 (194x)

Standard sample size calculations (theoretical n) are compared to the sample size requirements based on simulations at Level 2 and Level 3, as a variability of 0.1 to 1 is added to each of the four dose–response curve parameters where mu0 = 0.5 and sigma = 0.1. The variability refers to the varSlope, varEC50, varMaxResp, or varMinResp parameters in Formula (2), which serves to add variability to better model real-world experimental data. The relative increase in sample size requirements from standard calculations (theoretical n) are presented in parentheses for all sample sizes calculated using the simulation.

### Adding variability to Level 2 dose–response curve parameters reduces power and increases the required sample size

The above simulations took into account the variability of the *input* distributions for each parameter but did not add variability to the actual transformations. It is therefore not surprising that a fixed transformation based on dose–response curves might not greatly affect power because the inputs still map directly to the outputs and it is well-known that the *t*-test is robust to several types on non-normal distributions^10^. However, the rationale for this study is that translational research proceeds through a series of experiments, each adding variability. We hypothesized that power calculations based on the non-central *t* distribution that are typically used to guide investigators in sample size selection do not adequately account for the variability added by each of a sequence of experimental studies. To test this hypothesis, we performed simulations where variability was added to the transformations by introducing variability to the parameters of the dose–response curve.

#### Slope in dose–response curves

The slope parameter was varied by adding values from a normal distribution centered on 0 with a SD of 0.1, multiplied by the slope variability parameter. Introducing a small degree of variability to the slope parameter (e.g. 0.1) to the Level 2 simulation resulted in little appreciable change to the curve as compared to the baseline curve without any variability (Table 1). At a small effect size (difference between group means of 0.01 and sigma of 0.1), adding the variability of 0.1 to the slope parameter only increases the required sample size from 1571 to 1666, a 1.06x increase. Adding a variability of 0.5 to the slope parameter also at a group mean difference of 0.01 increases the sample size from 1571 to 2298, a 1.46x increase. Further increasing the slope variability to 1.0 changes the required sample size to 5743, a 3.65x increase. Similarly, when the group mean difference is increased to 0.11, a slope variability of 0.5 resulted in a 1.46x increase in sample size from 15 to 22, while a slope variability of 1.0 resulted in a 4.4x increase in sample size to 66.

These results demonstrate that as variability is added to the slope parameter of the dose–response function the sample size requirements increase, at a given power, alpha, group mean difference, and sigma. Changing sigma has a similar but reciprocal impact on sample size, as compared to changing the group mean difference (Fig. 3C), because the effect size is determined by the ratio of the two. At a sigma of 0.11, a slope variability of 1 increases the required sample size from 21 to 87, a 4.14x increase. With broader distributions in the simulated groups, e.g., sigma of 0.51, a slope variability of increases the sample size from 411 to 604, and a slope variability of 1 increases the sample size to 1838, a 4.47x increase. Increases in either slope variability or population sigma result in increased sample size requirements, and the larger the slope variability, the more apparent the effect of changing sigma on sample size.

#### EC_50_ in dose–response curves

**Figure 4:**
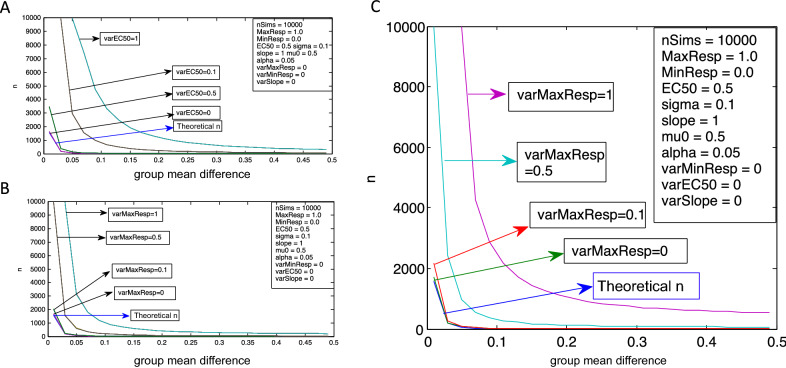
Effect of variability of (**A**) EC_50_ or (**B**) maximal response on sample size for two consecutive sigmoidal dose–response transformations with various group mean differences. The difference between the simulation sample size estimates (n) and the theoretical sample size increases at a given group mean difference when greater variability is added to the maximal response parameter, with alpha and power held constant. (**C**) Effect of variability of maximal response on sample size for three consecutive sigmoidal dose–response transformations with various group mean differences. The difference between the simulation sample size estimates and the theoretical sample size increases at a given group mean difference when a third dose–response function is added to the simulation, with variability in the maximal response parameter.

When variability was added to the EC_50_ parameter in the Level 2 simulation, there was a similar relationship as when variability was added to the slope parameter (Table 1; Fig. 4A). That is, the smaller the difference between group means (e.g. 0.01), the more substantial the increase in the required sample size as a result of adding variability to the dose–response relationship. Of the four parameters, increasing EC_50_ variability had the largest impact on sample size, with more than double the theoretical sample size required at a variability of 0.1 and > 10,000 at a variability of 0.5 or greater (Table 1).

#### Maximal and minimal responses in dose–response curves

The effect on sample size of adding variance to the minimal response and maximal response parameters yielded largely the same results. As with the EC_50_ parameter, at a small group mean difference (0.01), the n required to detect an effect was > 10,000 with a maximal or minimal response variability of 0.5 or greater (Table 1; Fig. 4B).

### Increasing the number of dose–response transformations from Level 1 to Level 3 amplifies the effect of variability on the relationship between power and sample size

Adding a third dose–response transformation (Level 3) further increases the required sample size for a given power (Table 1). Figure 4C depicts the effects of adding variability to the maximal response parameter, but similar results were found with all four dose–response curve parameters, again with the EC50 parameter having the largest impact on sample size requirements.

#### Maximal response in dose–response curves

At a group mean difference of 0.01 and a SD of 0.1, increasing maximal response variability by a factor of 0.1 to all dose–response functions included in Level 3 resulted in a 1.38x increase in the sample size calculations from 1571 to 2181 (Fig. 4C). When the difference between group means is increased to 0.11, changing the maximal response parameter variability to 0.1, 0.5, or 1 resulted in sample sizes of 23, 267, and 2159, respectively (Table 1). This corresponds to sample sizes that are 1.5x, 18x, and 144x greater than the theoretical n of 15. Compared to the level 2 simulation, the most notable increase in sample size requirements at level 3 are seen when comparing *n* at the maximum single parameter variability of 1 (Table 1; varMaxResp = 1, Fig. 3B,C).

### Introducing variability to multiple dose–response curve parameters for Levels 1 through 4 leads to considerably higher sample size requirements

**Figure 5:**
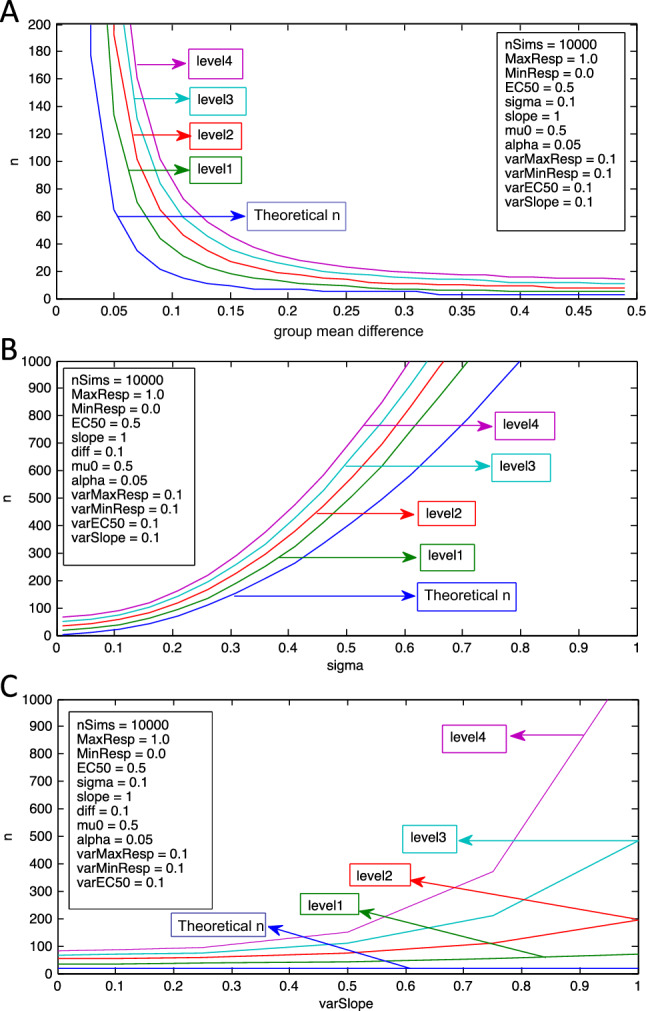
(**A**) Effect of increasing the number of dose–response levels on sample size when there is variability in all dose–response parameters. Depending on the difference in group means, the transformation of data through increasing levels of sigmoidal dose–response functions with fixed (0.1) variability in all four dose–response parameters (slope, EC_50_, maximal response, minimal response) resulted in simulation sample size requirements (n) that progressively increase, compared to standard sample size calculations (theoretical n). (**B**) Effect of increasing the number of dose–response levels on sample size at various sigma values. The sample size (n) derived by simulations using transformed dose–response data progressively increases from the theoretical sample size (theoretical n) as more dose–response levels are added to the simulation with fixed background variability (0.1) in all four curve parameters. The greater the number of dose–response levels in the simulation the greater the effect of background variability and dose–response transformations on the simulation sample sizes. (**C**) Effect of slope variability on sample size for various dose–response levels with fixed background dose–response curve variability. For a fixed group mean difference, the inclusion of fixed background variability (0.1) in EC_50_, maximal response, and minimal response amplifies the effect of slope variability on increasing simulation sample sizes (n) as compared to the theoretical sample sizes (theoretical n).

The final step in the simulation was to assess the combined effects of adding variability to all four parameters for Levels 1, 2, 3, and 4 (Fig. 5A). When minor (e.g., 0.1) variability is added to all four parameters for the dose–response transformations included in Level 4, the effects on sample size are comparable to adding a ten-fold higher degree of variability (e.g., 1.0) to a single curve parameter over fewer transformations (Levels 1–3). For example, for Level 4 at a group mean difference of 0.19 and adding a variability of 0.1 to all of the parameters, the required sample size changes from 7 to 32. In comparison, for the same group mean difference (0.19) but at Level 2, the variability of the slope parameter must be set 10 x higher, to 1.0, for the sample size to increase from 7 to 37 (Fig. 3B). These data support the previous conclusion that as dose–response transformations are added to the simulation, the sample size requirements increase to maintain the same power and alpha. At a group mean difference of 0.11 the theoretical sample size is 15. As the simulation complexity increases progressively from Level 1 to Level 2 to Level 3 to Level 4, with a variability of 0.1 applied to all four parameters, the sample sizes increase from 31 to 46 to 59 to 73. These values correspond to a 2.1x, 3.1x, 3.9x, and 4.9x increase in the required sample size compared to the theoretical sample size. Similar results were seen when changing sigma (Fig. 5B).

Increasing the variability in any one dose–response curve parameter with minimal background variability amongst the other parameters was also shown to substantially impact sample size requirements. When there is an existing baseline variability of 0.1 in the EC_50_, maximal response, and minimal response parameters, the impact of adding variability to the slope parameter is compounded. For Level 2, when a variability of 0.5 is added to the slope parameter alone, at a group mean difference of 0.11 there is a 1.5x increase from the theoretical sample size of 15 to 22 (Table 1). In comparison, for Level 2 with a background variability of 0.1 in the other three parameters (Fig. 5C), increasing the slope variability to 0.5 results in a 4.3x increase in the required sample size (n = 73) at a comparable group mean difference of 0.1. Adding another two dose–response transformations to the simulation (i.e. Level 4) at a group mean difference of 0.1 further increases the sample size requirements to 152, an 8.9x increase as compared to the theoretical n.

Finally, a counterintuitive finding depicted in Fig. 5 is that the effect of nesting on spreading of experimental data becomes lower as the depth of nesting increases. For example, with a variability of 0.1 added to all four parameters, there is a 112% increase in sample size requirements moving from theoretical sample size calculations to Level 1 calculations, at a group mean difference of 0.1. With adding subsequent levels of nesting, even with the propagation of parameter variability, the relative increase becomes smaller as each higher Level is reached, e.g. there is a 50% increase moving from Level 1 to Level 2, a 28% increase moving from Level 2 to Level 3, and 25% increase moving from Level 3 to Level 4.

## Discussion

The Princess and the Pea problem gives a name to a specific challenge of translational research, which while well-understood, has not been quantified until now. The pathway from preclinical research to clinical trials includes an inherent spread of experimental variability. Despite a conceptual recognition of this accumulation of variability, the standard methods used by investigators to predict an appropriate sample size do not incorporate this spread of experimental data. Moreover, pure analytical or theoretical approaches to address the Princess and the Pea problem are limited by the fact that most statistical approaches, from which a theoretical analysis would be generated, almost inevitably are based on data that violates necessary assumptions^11^. A Monte Carlo simulation was therefore used to take advantage of a typical sigmoidal dose–response relationship while also accounting for potential assumption violations to best quantify the effects of experimental variability on power and sample size calculations.

As seen in Fig. 2, the process of transforming study data through nested dose–response functions, akin to transitioning through sequential studies in a drug development program, increases the spread of variance in the experimental data, making it harder to identify a clinical effect.

When small amounts of “real-world” variability are added to the input parameters of the simulated experiments, there is even greater widening of the distributions. In terms of study design and planning, these results suggest that accounting for the spread of variability in translational research necessitates a drastic increase in sample sizes in order to maintain a desired power and alpha. At Level 2, used to simulate two consecutive studies, the variability for a single parameter must be set high (e.g., an increase in variability from 0 to 1) to result in an appreciable change in sample size requirements. However, as more dose–response curve transformations are introduced into the simulation (modeling more steps between preclinical research and clinical trials), less dose–response parameter variability is needed to see similar increases in sample size requirements. When minor variability is added to all four curve parameters for the Level 4 simulation (approximating the variability encountered in the drug development process), there is a drastic increase in sample size requirements, e.g. a 4.9x increase in sample size with a variability of 0.11 in the four parameters. When a larger variability is added to a single parameter, with a background of minor variability amongst other parameters, there is again a substantial jump in the required sample size when compared to standard theoretical power and sample size calculations. Between the four parameters, variability in EC_50_ appears to have the greatest impact on sample size, followed by maximal and minimal response, and then slope.

This simulation quantifies just how large sample size requirements become for a desired power when the accumulation of variability that occurs during translational research programs is taken into account***.*** For example, even at Level 2 of 4, with a group mean difference of 0.01 and SD of 0.1, introducing a variability of 0.5 to the EC_50_ parameter alone increases the sample size requirements from a reasonable target of 1571 subjects to a potentially prohibitive sample size greater than 10,000 (Table 1). Even with larger effect sizes and correspondingly smaller initial sample size requirements, small changes in dose–response curve input and output variability substantially impact sample size and the ability to detect an effect. Power calculations based on the non-central *t* distribution are often used to guide investigators in sample size selections, but do not adequately account for the variability accumulated over a sequence of experimental studies. If the sample size used for a trial based on standard power calculations is 15 while this simulation finds a minimum sample size of 73 at Level 4 (Fig. 5A), it should come as no surprise that researchers frequently fail to detect a significant effect at the clinical trial level (Fig. 5A). In reality, trials are typically limited to hundreds (e.g., Phase 2) to a few thousand (e.g., Phase 3) of participants. Working within the bounds of a feasible clinical trial, these results suggest that the ability to detect a significant difference between experimental groups is reduced by the inevitable spread of variance in translational research. Clinical trials for rare diseases exemplify the importance of selecting adequate and feasible sample size targets. Given the inherent rarity of these diseases, setting recruitment targets that are sufficient to detect a difference between experimental groups while also feasible is of central importance^12^. When hundreds, rather than thousands, of patients are recruited for Phase 3 trials, the aforementioned spread of variance can easily mask the effect of a therapy and result in a failed trial. In this context, increasing sample size numbers to overcome variability is frequently not feasible. Even in larger trials, where thousands of patients can be recruited, this same challenge can lead to the dissipation of a clinically meaningful effect within the noise of study variability.

When planning a clinical trial, investigators can typically predict the variability of study measurements but not of the effect size. With this simulation, the measured effect size in an animal model can effectively be translated to a predicted effect size for a clinical trial by taking into account the variability accumulated with sequential experimental steps. For instance, if a study drug is found to protect 50% of retinal ganglion cells (RGCs) from dying in a rat model of glaucoma, investigators can predict that it also protects 50% of human RGCs from dying. The effect size, however, will almost inevitably be reduced at the human trial level due to the greater variability encountered in human populations. Rather than relying on assumptions that a preclinical effect size (i.e. effect size in an animal model) will remain the same throughout the translational process, investigators can use tools derived from the simulations outlined in this study to better predict the effect size of a given drug once at the clinical trial stage.

The results of these simulations also suggest some approaches to prevent translational failure as a result of the Princess and The Pea problem. We propose that in order to design successful translational studies of novel therapeutic classes, investigators should aim for more robust preclinical effect sizes than routine power analyses might suggest. Smaller preclinical effect sizes may not warrant progression to larger trials once investigators account for the variability inherent to the study design and to the therapeutic effect. Given that effect size depends on the ratio between the group mean difference and the population standard deviation, investigators must simultaneously seek larger differences between experimental groups and smaller variation in their data to better design translational studies.

Scrupulous control of sources of variability throughout the translational research process can also help mitigate some of these effects. From the way investigators collect data and record measurements to the way they culture cell lines and interact with experimental animals, investigators can actively seek ways of minimizing variability within their experimental systems, recognizing how this variability will propagate, as simulated in this study by carrying forward variability through each Level. The actual effect of a therapy will itself have a degree of variability that cannot be directly controlled by the careful planning and execution of studies. Some spreading of variance is therefore an unavoidable consequence of developing new therapies and of transitioning between experimental systems.

Given that each sequential dose–response transformation, or Level, (i.e. “mattress”) resulted in the spreading of a given effect size (i.e. “pea”) due to variability, we also suggest designing translational drug development programs with fewer steps, where possible. If the number of steps between initial preclinical biochemical studies and eventual human clinical trials can be reduced without compromising study integrity, it may be possible to more accurately predict the propagation of the true effect size and calculate accurate sample size requirements.

Another helpful tool to mitigate the effect of spreading experimental variability is to use biomarkers throughout the translational research process. Biomarkers play an important role in bridging laboratory and human studies. For instance, biomarkers can provide subject-specific biologic data on variations that influence a given drug’s efficacy and toxicity^13^. Assessing the same biomarker at each experimental level can help to control for the loss of the ability to detect an effect size given that the measured outcome often varies at each stage (e.g., fraction bound in a receptor-binding assay versus visual acuity letter score in a clinical trial).

The modeling used in the present simulation made certain assumptions. When multiple dose–response curve transformations were included in the simulation, the variability added to a given curve parameter was the same for each experimental level. In reality, we are unlikely see an exact propagation of the same variance for an experimental parameter. We may in fact expect the variability to increase for a given parameter as the study progresses to increasingly more complex biological systems. This study was designed using a sequence of sigmoidal dose–response curves as an abstraction for the steps involved in the development of a new therapy. The choice of a dose–response curve has a number of implications for our results. For one, what we measure as a response outcome may change as we move between experimental systems, which was not accounted for in our simulation. Our conclusions are also limited to studies dealing with data that follows the feature of a dose–response transformation. The dose–response curve has the features of asymptotes to a maximum, a minimum, a half-maximal response, and a slope. Other curves grow exponentially with discrete intervals of time, as in a typical exponential curve, or with multiple phases and rates of growth, as in biological growth curves. We can in future investigate whether similar effects are seen using other relationships that exist between experimental data.

The simulation also assumed normal distributions for all datasets. Given that many biological functions do not have a normal distribution, this assumption could result in under- or overestimation of the spread of variance. The selection of an appropriate test for a given dataset is of critical importance for all research^14^. Monte Carlo simulations, as used by our simulation, are particularly useful when analyzing data that do not conform to the assumptions required for a given statistical test^15^. Examination of Type I error rate, the probability of rejecting a false null hypothesis and power estimates, for instance, can be appropriately assessed using Monte Carlo methods^15^.

In summary, the Princess and the Pea problem is a challenge inherent to translational research, where an effect seen early in the development process becomes difficult to detect at the clinical trial level due to a gradual accumulation of variance with each experimental step. Consequently, large sample sizes are needed to detect an effect that may have appeared promising early in the development process. This study provides a quantitative rationale for the frequent failure of translational research, namely the fairy tale-like assumption that an effect size is equally detectable at all levels of translation, despite the spread of variability. The failure to account for the increase in variability during the translational process can lead to an underestimation of sample sizes for later clinical studies. The simulations in this study can be used as tools to better account for the spread of experimental variability when making power and sample size calculations and, ultimately, to design translational studies that are both feasible and successful.

## Methods

### Monte Carlo simulation

Monte Carlo simulations were used to quantify the effects of variability on individual experiments and on a series of consecutive experiments. For this study we selected a sigmoidal dose–response curve as the basis of the simulated experiments based on its relevance to a variety of biomedical processes. These simulations were designed to better account for the propagation of variability in translational research, which is not typically considered in standard power and sample size calculations.

Functions to simulate power and sample size calculations were written in MATLAB (MathWorks, Natick, MA), based on an unpaired equal-variance *t*-test (“simulation n”). The MATLAB *randn* function was used to generate randomly generated numbers from a normal distribution and two distributions were generated (Group A, Group B) with a defined difference between group means (mu0; mu0 = 0.5 for all simulations where mu0 was held constant). The *randn* function is robust and is based on the Mersenne twister algorithm, with a period of 2^19937^ − 1. The ratio of significant to non-significant t-statistics for 10,000 runs of the simulation with various sample sizes determined the power for the simulation (see Supplementary Material).

To confirm that in the absence of variability our simulation replicated standard functions, we compared the sample size requirements for a given power using our simulation to the standalone MATLAB *sampsizepwr* function. After confirming that our simulation produced reliable sample size estimates, a dose–response relationship was introduced into the model. The simulation was designed to include a maximum of four dose–response transformations, or levels (Fig. 1). The Level 0 simulation replicates a typical unpaired equal-variance *t*-test with a given difference between the two experimental group means, i.e. no transformation of the data. The Level 1 simulation transforms drug doses for both groups by a dose–response function and again runs a t-test. Level 2 uses the response output values from Level 1 as the input for a second dose–response transformation of both groups. Level 3, similarly, uses the response output from Level 2 and transforms these data again by the same dose–response function. Finally, the Level 4 simulation uses the Level 3 response output as the dose input for another dose–response transformation. In other words, each level reflects progressively greater numbers of nested dose–response curves, using the output of one level as input to the next, thus modeling a series of biological steps in the action of a drug at biochemical, biological, and clinical levels.

The model was designed to allow for variability in four dose–response curve input parameters: the half maximal effective concentration (EC_50_), the slope of the dose–response curve (slope), the maximal response (MaxResp), and the minimal response (MinResp).

The power and sample size calculations in this study are based on the ability of the simulation to detect a clinical effect in the simulated experiment(s). The study was designed to simulate up to a maximum of 10,000 trials, generating a *t*-statistic for each of the 10,000 runs, as mentioned above. If a single run was significant, based on an alpha of 0.05, the trial was coded as a 1. Conversely, if the *t*-statistic for the simulated trial was non-significant, the run was coded as a 0. The ratio of significant (1) to non-significant (0) trials then determined the power of the simulation to detect an effect for the parameters and variability of a given hypothetical clinical trial.

### Source of model parameters

In order to populate numbers for the main parameters of the simulation, namely EC_50_, slope, maximal response, minimal response, group A drug dose, and group B drug dose a vector of random inputs was generated for each. For example, the input for group B drug dose was generated as follows:

1 $$b=\left[\sigma \times randn\left(n,1\right)\right]+\mu +\Delta $$

where $b$ = vector for adding variability to the drug dose input for group B, $\sigma$ = standard deviation (SD) of drug dose, $randn\left(n,1\right)$ = n-by-1 column vector of normally distributed random numbers with mean 0 and variance 1, μ = arithmetic mean of group A drug dose, Δ = difference between group means.

Variability was added to the dose–response curve parameters using a similar function as the one above, with a modifiable variability parameter. For example, the slope for the dose–response curve was modeled as follows:

2 $$simSlope=Slope+\left[randn\left(n,1\right)\times varSlope\right]$$

where $simSlope$ = slope parameter that will be used for the dose–response function, $Slope$ = slope parameter before adding variability, $randn\left(n,1\right)$ = n-by-1 column vector of normally distributed random numbers with mean 0 and variance 1, $varSlope$ = amount of variability to add to the slope.

A dose–response curve was modeled using the following equation:

3 $$Response=\frac{MaxResp-MinResp}{1+{10}^{(\mathrm{log}{EC}_{50}-\mathrm{log}Dose)\times Slope}}$$

where, *Dose* = drug concentration (group A or group B), *MaxResp* = maximal response, *MinResp* = minimal response, *EC*_*50*_ = half-maximal concentration, *Slope* = slope of dose-response curve, *Response* = ${\overline{x} }_{A}\mathrm{or} {\overline{x} }_{B}$.

An unpaired equal-variance *t*-test was used to compare differences between the two normally distributed groups. This test was selected because of its utility in power calculations^16^.

All sample sizes refer to the sample size per group. “Theoretical n” refers to the sample size calculated using standard MATLAB methods (namely, *sampsizepwr)* whereas “simulation n” refers to the sample size calculated using the Monte Carlo simulation described above. The calculations in this study assume equal group sizes; however, the simulation was designed to allow for the use of unequal group sizes, if desired.

## Supplementary Information

Supplementary Information.
