# Supplementary material for: A quantitative approach to the spread of variance in translational research using Monte Carlo simulation
Source: Sci Rep. 2022 Apr 15;12:6274. doi: 10.1038/s41598-022-09921-3 (PMC9012853; doi:10.1038/s41598-022-09921-3)
Supplement: Supplementary file 1 — Supplementary Information. [file 41598_2022_9921_MOESM1_ESM.pdf]

doseResponsePwr

```
function power = doseResponsePwr(nSims,n,MaxResp,MinResp,EC50,slope,diff,sigma,mu0,alpha)
global TBYDF;
if isempty(n) | isempty(alpha)
    power = [];
    return
end
clear h a b hfast
a = zeros(n,1);
b = zeros(n,1);
h = zeros(1,nSims);
hfast = zeros(1,nSims);
respa = zeros(n,1);
respb = zeros(n,1);
logEC50 = log(EC50);
nx = n; %The code can be used for unequal sample sizes in the future
ny = n;
dfe = nx + ny - 2;
for i=1:nSims
    a=(sigma .* randn(n,1)) + mu0;
    b=(sigma .* randn(n,1))+ mu0 + diff;
    respa = MinResp + (MaxResp-MinResp)./(1+10.^((logEC50-log(max(a,0.001))).*slope));
    respb = MinResp + (MaxResp-MinResp)./(1+10.^((logEC50-log(max(b,0.001))).*slope));
    meana = sum(respa)./nx;
    meanb = sum(respb)./ny;
    difference = abs(meana - meanb);
    s2x =(sum(respa.^2)- nx * meana^2)/(nx-1);
    s2y =(sum(respb.^2)- ny * meanb^2)/(ny-1);
    sPooled = sqrt(((nx-1) .* s2x + (ny-1) .* s2y) ./ dfe);
    se = sPooled .* sqrt(1./nx + 1./ny);
    ratio = difference ./ se;
    hfast(i) = ratio > TBYDF(dfe);
end
power = mean(hfast);
```

doseResponsePwrLevel0

```
function power = doseResponsePwrLevel0(nSims,n,MaxResp,MinResp,EC50,slope,varMaxResp,varMinResp,varEC50,varSlope,diff,sigma,mu0,alpha)

global TBYDF;
if isempty(n) || isempty(alpha)
    power = [];
    return
end
clear h a b hfast
a = zeros(n,1);
b = zeros(n,1);
h = zeros(1,nSims);
hfast = zeros(1,nSims);
respa = zeros(n,1);
respb = zeros(n,1);
logEC50 = log(EC50);
nx = n; %The code can be used for unequal sample sizes in the future
ny = n;
dfe = nx + ny - 2;
for i=1:nSims
    simLogEC50 = log(max((EC50+randn(n,1).* varEC50),0.01));
```

```
    simSlope = slope+randn(n,1).* varSlope;
    simMaxResp = MaxResp+randn(n,1).* varMaxResp;
    simMinResp = MinResp+randn(n,1).* varMinResp;
    simLogEC502 = log(max((EC50+randn(n,1).* varEC50),0.01));
    simSlope2 = slope+randn(n,1).* varSlope;
    simMaxResp2 = MaxResp+randn(n,1).* varMaxResp;
    simMinResp2 = MinResp+randn(n,1).* varMinResp;
    a=(sigma .* randn(n,1)) + mu0;
    b=(sigma .* randn(n,1))+ mu0 + diff;
    meana = sum(a)./nx;
    meanb = sum(b)./ny;
    difference = abs(meana - meanb);
    s2x =(sum(a.^2)- nx * meana^2)/(nx-1);
    s2y =(sum(b.^2)- ny * meanb^2)/(ny-1);
    sPooled = sqrt(((nx-1) .* s2x + (ny-1) .* s2y) ./ dfe);
    se = sPooled .* sqrt(1./nx + 1./ny);
    ratio = difference ./ se;
    hfast(i) = ratio > TBYDF(dfe);
end
power = mean(hfast);
```

searchDoseRespN

```
function N=searchDoseRespN(nSims,desiredpower,lohi,MaxResp,MinResp,EC50,slope,diff,sigma,mu0,alpha)
%searchbinaryN Sample size calculation via binary search

nlo = repmat(lohi(1),size(alpha));  % guaranteed lower bound
nhi = repmat(lohi(2),size(alpha));  % trial upper bound
%obspower = F(p0,p1,alpha,tail,nhi);
obspower = doseResponsePwr(nSims,nhi,MaxResp,MinResp,EC50,slope,diff,sigma,mu0,alpha);

% Iterate on n until we achieve the desired power
todo = 1:numel(alpha);
while(~isempty(todo))
    % Find an upper bound for the required sample size
    todo = todo(obspower(todo)<desiredpower(todo));
    nhi(todo) = nhi(todo) * 2;
    if sum(nhi(todo)) > 10000
        print('Impossible to reach power')
    end
    obspower(todo) = doseResponsePwr(nSims,nhi(todo),MaxResp,MinResp,EC50,slope,diff,sigma,mu0,alpha(todo));
end
% Now nhi is a guaranteed upper bound

% Binary search between these bounds for required sample size
todo = find(nhi > nlo+1);
while(~isempty(todo))
    n = floor((nhi(todo)+nlo(todo))/2);
    obspower = doseResponsePwr(nSims,n,MaxResp,MinResp,EC50,slope,diff,sigma,mu0,alpha(todo));
    toohigh = (obspower>desiredpower(todo));
    nhi(todo(toohigh)) = n(toohigh);
    nlo(todo(~toohigh)) = n(~toohigh);
    todo = todo(nhi(todo)>nlo(todo)+1);
end
N = nhi;
end
```

doseResponsePwrVar

```
function power = doseResponsePwrVar(nSims,n,MaxResp,MinResp,EC50,slope,varMaxResp,varMinResp,varEC50,varSlope,diff,sigma,mu0,alpha)

global TBYDF;
if isempty(n) || isempty(alpha)
    power = [];
    return
end
clear h a b hfast
a = zeros(n,1);
b = zeros(n,1);
h = zeros(1,nSims);
hfast = zeros(1,nSims);
respa = zeros(n,1);
respb = zeros(n,1);
logEC50 = log(EC50);
nx = n; %The code can be used for unequal sample sizes in the future
ny = n;
dfe = nx + ny - 2;
for i=1:nSims
    simLogEC50 = log(max((EC50+randn(n,1).* varEC50),0.01));
    simSlope = slope+randn(n,1).* varSlope;
    simMaxResp = MaxResp+randn(n,1).* varMaxResp;
    simMinResp = MinResp+randn(n,1).* varMinResp;
    a=(sigma .* randn(n,1)) + mu0;
    b=(sigma .* randn(n,1))+ mu0 + diff;
    respa = simMinResp + (simMaxResp-simMinResp)./(1+10.^((simLogEC50-log(max(a,0.001))).*simSlope));
    respb = simMinResp + (simMaxResp-simMinResp)./(1+10.^((simLogEC50-log(max(b,0.001))).*simSlope));
    meana = sum(respa)./nx;
    meanb = sum(respb)./ny;
    difference = abs(meana - meanb);
    s2x =(sum(respa.^2)- nx * meana^2)/(nx-1);
    s2y =(sum(respb.^2)- ny * meanb^2)/(ny-1);
    sPooled = sqrt(((nx-1) .* s2x + (ny-1) .* s2y) ./ dfe);
    se = sPooled .* sqrt(1./nx + 1./ny);
    ratio = difference ./ se;
    hfast(i) = ratio > TBYDF(dfe);
end
power = mean(hfast);
```

searchDoseRespNVar

```
function N=searchDoseRespNVar(nSims,desiredpower,lohi,MaxResp,MinResp,EC50,slope,varMaxResp,varMinResp,varEC50,varSlope,diff,sigma,mu0,alpha)
%searchbinaryN Sample size calculation via binary search

nlo = repmat(lohi(1),size(alpha));  % guaranteed lower bound
nhi = repmat(lohi(2),size(alpha));  % trial upper bound
obspower = doseResponsePwrVar(nSims,nhi,MaxResp,MinResp,EC50,slope,varMaxResp,varMinResp,varEC50,varSlope,diff,sigma,mu0,alpha);

% Iterate on n until we achieve the desired power
todo = 1:numel(alpha);
while(~isempty(todo))
    % Find an upper bound for the required sample size
    todo = todo(obspower(todo)<desiredpower(todo));
    nhi(todo) = nhi(todo) * 2;
    if sum(nhi(todo)) > 10000
       N = 10001;
```

```
        return
    end
    obspower(todo) =
doseResponsePwrVar(nSims,nhi(todo),MaxResp,MinResp,EC50,slope,varMaxResp,varMinResp,varEC50,varSlope,diff,sigma,mu0,alpha(todo));
end
% Now nhi is a guaranteed upper bound

% Binary search between these bounds for required sample size
todo = find(nhi > nlo+1);
while(~isempty(todo))
    n = floor((nhi(todo)+nlo(todo))/2);
    obspower = doseResponsePwrVar(nSims,n,MaxResp,MinResp,EC50,slope,varMaxResp,varMinResp,varEC50,varSlope,diff,sigma,mu0,alpha(todo));
    toohigh = (obspower>desiredpower(todo));
    nhi(todo(toohigh)) = n(toohigh);
    nlo(todo(~toohigh)) = n(~toohigh);
    todo = todo(nhi(todo)>nlo(todo)+1);
end
N = nhi;
End
```

doseResponsePwrLevel2

```
function power = doseResponsePwrLevel2(nSims,n,MaxResp,MinResp,EC50,slope,varMaxResp,varMinResp,varEC50,varSlope,diff,sigma,mu0,alpha)

global TBYDF;
if isempty(n) || isempty(alpha)
    power = [];
    return
end
clear h a b hfast
a = zeros(n,1);
b = zeros(n,1);
h = zeros(1,nSims);
hfast = zeros(1,nSims);
respa = zeros(n,1);
respb = zeros(n,1);
logEC50 = log(EC50);
nx = n; %The code can be used for unequal sample sizes in the future
ny = n;
dfe = nx + ny - 2;
for i=1:nSims
    simLogEC50 = log(max((EC50+randn(n,1).* varEC50),0.01));
    simSlope = slope+randn(n,1).* varSlope;
    simMaxResp = MaxResp+randn(n,1).* varMaxResp;
    simMinResp = MinResp+randn(n,1).* varMinResp;
    simLogEC502 = log(max((EC50+randn(n,1).* varEC50),0.01));
    simSlope2 = slope+randn(n,1).* varSlope;
    simMaxResp2 = MaxResp+randn(n,1).* varMaxResp;
    simMinResp2 = MinResp+randn(n,1).* varMinResp;
    a=(sigma .* randn(n,1)) + mu0;
    b=(sigma .* randn(n,1))+ mu0 + diff;
    respa = simMinResp + (simMaxResp-simMinResp)./(1+10.^((simLogEC50-log(max(a,0.001))).*simSlope));
    respb = simMinResp + (simMaxResp-simMinResp)./(1+10.^((simLogEC50-log(max(b,0.001))).*simSlope));
    respa2 = simMinResp2 + (simMaxResp2-simMinResp2)./(1+10.^((simLogEC502-log(max(respa,0.001))).*simSlope2));
    respb2 = simMinResp2 + (simMaxResp2-simMinResp2)./(1+10.^((simLogEC502-log(max(respb,0.001))).*simSlope2));
    meana = sum(respa2)./nx;
    meanb = sum(respb2)./ny;
    difference = abs(meana - meanb);
    s2x =(sum(respa2.^2)- nx * meana^2)/(nx-1);
    s2y =(sum(respb2.^2)- ny * meanb^2)/(ny-1);
```

```
    sPooled = sqrt(((nx-1) .* s2x + (ny-1) .* s2y) ./ dfe);
    se = sPooled .* sqrt(1./nx + 1./ny);
    ratio = difference ./ se;
    hfast(i) = ratio > TBYDF(dfe);
end
power = mean(hfast);
```

searchDoseRespNLevel2

```
% Note that the searchDoseRespNLevelX is the same for levels 3, 4, and if desired, higher
% with the only difference being that doseResponsePwrLevelX must be called
function N=searchDoseRespNLevel2(nSims,desiredpower,lohi,MaxResp,MinResp,EC50,slope,varMaxResp,varMinResp,varEC50,varSlope,diff,sigma,mu0,alpha)
%searchbinaryN Sample size calculation via binary search

nlo = repmat(lohi(1),size(alpha));  % guaranteed lower bound
nhi = repmat(lohi(2),size(alpha));  % trial upper bound
obspower = doseResponsePwrLevel2(nSims,nhi,MaxResp,MinResp,EC50,slope,varMaxResp,varMinResp,varEC50,varSlope,diff,sigma,mu0,alpha);

% Iterate on n until we achieve the desired power
todo = 1:numel(alpha);
while(~isempty(todo))
    % Find an upper bound for the required sample size
    todo = todo(obspower(todo)<desiredpower(todo));
    nhi(todo) = nhi(todo) * 2;
    if sum(nhi(todo)) > 10000
       N = 10001;
        return
    end
    obspower(todo) =
doseResponsePwrLevel2(nSims,nhi(todo),MaxResp,MinResp,EC50,slope,varMaxResp,varMinResp,varEC50,varSlope,diff,sigma,mu0,alpha(todo));
end
% Now nhi is a guaranteed upper bound

% Binary search between these bounds for required sample size
todo = find(nhi > nlo+1);
while(~isempty(todo))
    n = floor((nhi(todo)+nlo(todo))/2);
    obspower = doseResponsePwrLevel2(nSims,n,MaxResp,MinResp,EC50,slope,varMaxResp,varMinResp,varEC50,varSlope,diff,sigma,mu0,alpha(todo));
    toohigh = (obspower>desiredpower(todo));
    nhi(todo(toohigh)) = n(toohigh);
    nlo(todo(~toohigh)) = n(~toohigh);
    todo = todo(nhi(todo)>nlo(todo)+1);
end
N = nhi;
end
```

doseresponselevel3bydiffvarMinResp

```
clear
nSims = 10000;
maxN = 10000;
MaxResp = 1.0;
MinResp = 0.0;
EC50 = 0.5;
sigma = 0.1;
slope = 1;
```

```
%diff = 0.1;
mu0 = 0.5;
alpha = 0.05;
varMaxResp = 0;
%varMinResp = 0;
varEC50 = 0;
varSlope = 0;
global TBYDF;
TBYDF = tinv(1-alpha/2,1:maxN*2);
results = zeros(maxN,3);
rowNumber = 1;
for diff = 0.01:0.02:0.5
    for varMinResp = [0 0.1 0.5 1]
    nBasedOnCalc = sampsizepwr('t',[mu0 sigma],mu0+diff,[0.8],[]) * 2 - 3
    nBasedOnSim = searchDoseRespNLevel3(nSims,0.8,[2 nBasedOnCalc *
2],MaxResp,MinResp,EC50,slope,varMaxResp,varMinResp,varEC50,varSlope,diff,sigma,mu0,alpha)
    results(rowNumber,1) = diff;
    results(rowNumber,2) = nBasedOnSim;
    results(rowNumber,3) = nBasedOnCalc;
    rowNumber = rowNumber + 1;
    end
end
xlswrite('doseresponseLevel3V1ResultsByDiffvarMinRespAug162013Expt1.xls',results)
```

Doseresponsealllevelbyvarslope

```
clear
nSims = 10000;
maxN = 10000;
MaxResp = 1.0;
MinResp = 0.0;
EC50 = 0.5;
sigma = 0.1;
slope = 1;
diff = 0.1;
mu0 = 0.5;
alpha = 0.05;
varMaxResp = 0.1;
varMinResp = 0.1;
varEC50 = 0.1;
%varSlope = 0.1;
global TBYDF;
TBYDF = tinv(1-alpha/2,1:maxN*2);
results = zeros(maxN,3);
rowNumber = 1;
if matlabpool('size') == 0 % checking to see if my pool is already open
    matlabpool open 4
end
for varSlope = [0 0.1 0.25 0.5 0.75 1]
    nBasedOnCalc = sampsizepwr('t',[mu0 sigma],mu0+diff,[0.8],[]) * 2 - 3
    nBasedOnSim4 = searchDoseRespNLevel4(nSims,0.8,[2 nBasedOnCalc *
2],MaxResp,MinResp,EC50,slope,varMaxResp,varMinResp,varEC50,varSlope,diff,sigma,mu0,alpha)
    nBasedOnSim3 = searchDoseRespNLevel3(nSims,0.8,[2 nBasedOnCalc *
2],MaxResp,MinResp,EC50,slope,varMaxResp,varMinResp,varEC50,varSlope,diff,sigma,mu0,alpha)
    nBasedOnSim2 = searchDoseRespNLevel2(nSims,0.8,[2 nBasedOnCalc *
2],MaxResp,MinResp,EC50,slope,varMaxResp,varMinResp,varEC50,varSlope,diff,sigma,mu0,alpha)
    nBasedOnSim = searchDoseRespNVar(nSims,0.8,[2 nBasedOnCalc *
2],MaxResp,MinResp,EC50,slope,varMaxResp,varMinResp,varEC50,varSlope,diff,sigma,mu0,alpha)
```

```
    results(rowNumber,1) = varSlope;
    results(rowNumber,2) = nBasedOnCalc;
    results(rowNumber,3) = nBasedOnSim;
    results(rowNumber,4) = nBasedOnSim2;
    results(rowNumber,5) = nBasedOnSim3;
    results(rowNumber,6) = nBasedOnSim4;
    rowNumber = rowNumber + 1;
end
```
